# Feasibility of CardioSecur®, a Mobile 4-Electrode/22-Lead ECG Device, in the Prehospital Emergency Setting

**DOI:** 10.3389/fcvm.2020.551796

**Published:** 2020-10-09

**Authors:** Sebastian Spaich, Hanna Kern, Thomas A. Zelniker, Jan Stiepak, Michael Gabel, Erik Popp, Hugo A. Katus, Michael R. Preusch

**Affiliations:** ^1^Department of Cardiology, Angiology, and Pneumology, University Hospital Heidelberg, Heidelberg, Germany; ^2^Department of Cardiology, Robert-Bosch-Krankenhaus, Stuttgart, Germany; ^3^Department of Anaesthesiology, University Hospital Heidelberg, Heidelberg, Germany; ^4^Division of Cardiology, Medical University of Vienna, Vienna, Austria; ^5^Institute of Medical Biometry and Informatics, University of Heidelberg, Heidelberg, Germany

**Keywords:** acute coronary syndrome, electrocardiogram, ECG, STEMI, myocardial infarction, user-friendliness, feasibility

## Abstract

**Background:** This study explores the application of CardioSecur® (CS-ECG), a hand-held 4-electrode/22-lead ECG-device, in comparison with conventional 12-lead electrocardiogram (c12L-ECG) in patients with acute chest pain in the prehospital emergency setting.

**Methods:** CS-ECG systems were provided for two physician-staffed emergency ambulances and parallel recordings of c12L-ECG and CS-ECG were obtained from all patients with acute chest pain. Treating emergency physicians were asked to evaluate the CS-ECG system with a standardized questionnaire. Following study completion, acquired ECGs were analyzed separately by two independent cardiologists blinded to all other medical records.

**Results:** Over a period of 20 months a total of 203 patients were included in our study. According to a standardized questionnaire, 79% of emergency medical professionals preferred application of CS-ECG, with 87% of teams judging CS-ECG to be beneficial for patients. Morover, 79% of physicians reported a reduction in time to definitive diagnosis with implementation of CS-ECG. The majority of professional users attested user-friendliness and feasibility of CS-ECG in terms of easy general handling (94%), application (93%), and placement of electrodes (98%). During prehospital triage, both c12L-ECG and CS-ECG correctly identified 31 (91%) patients with ST-elevation myocardial infarction (STEMI).

**Conclusion:** In this first pilot study, implementation of the CardioSecur®-ECG system in the prehospital emergency setting demonstrated feasibility and user-friendliness so that emergency teams generally preferred CS-ECG to c12L-ECG. Diagnostic yield of CS-ECG was similar to c12L-ECG recordings.

## Introduction

Acute chest pain is considered one of the most prevalent symptoms that cause patients to initiate emergency medical contact in developed countries (1–3). Acute coronary syndrome (ACS), as one of the most frequent and potentially life-threatening causes of acute chest pain, needs to be identified as soon as possible, since early recognition has significant impact on management and prognosis (2–6). However, prehospital diagnostic tools to properly assess patients with acute chest pain in general and ACS in particular are very limited. Generally, prehospital diagnostic algorithms for acute chest pain heavily rely on patient history, physical examination and subsequent clinical judgement of treating physicians (2, 7, 8). When ACS is suspected performing a 12-lead electrocardiogram (ECG) is the only recommended prehospital tool with instant and broad availability (2, 9). These ECG recordings have paramount implications for risk stratification and treatment of ACS patients including timing of cardiac catheterization (2, 8, 10). Therefore, a conventional 12-lead electrocardiogram (c12L-ECG) serves as a critical pillar of prehospital triage and management of suspected ACS. However, recording of a c12L-ECG requires placement of 10 electrodes at prespecified anatomic landmarks and may even have to be augmented by additional electrodes to enable assessment of right ventricular and posterior ischemia (2). While c12L-ECG acquisition is essential for prehospital triage of ACS patients, electrode placement can be both time-consuming and challenging in terms of correct positioning in the prehospital emergency setting. In this regard, studies demonstrate c12L-ECG acquisition to be prone to incorrect placement—even by medical professionals in non-emergency settings—and proper regular training to be necessary for correct placement and recording (11, 12).

With development of CardioSecur^®^ (CS), a mobile ECG-device is available that requires placement of only four electrodes at easily accessible positions to generate a total of 22 leads so that time-consuming and error-prone placement of electrodes to obtain additional leads (as recommended for diagnosis/exclusion of strictly posterior myocardial infarction) is no longer necessary. We hypothesized that this simplification might be helpful to accelerate and optimize prehospital management of ACS patients. At the same time, the additional leads may provide improved diagnostic yield and add precision in early triage.

Therefore, this pilot study explores the prehospital value of CardioSecur^®^ (CS-ECG) in comparison to gold standard c12L-ECG, providing preliminary data on feasibility, diagnostic yield and safety, as well as user-friendliness and patient comfort of CS-ECG recordings in the prehospital emergency setting.

## Methods

### Study Design and Setting

Over a period of 20 months (May 2016–December 2017), two physician-staffed emergency ambulances in Heidelberg, Germany were equipped with the CardioSecur^®^ (CS) system (Personal MedSystems GmbH) in addition to the c12L-ECG devices normally provided. The CS technology and system derive from the vector loop concept and are based on the original EASI lead system and its enhancements (13–19). CardioSecur^®^ has been approved as a class IIa medical product with its CE mark valid throughout Europe.

**Figure 1:**
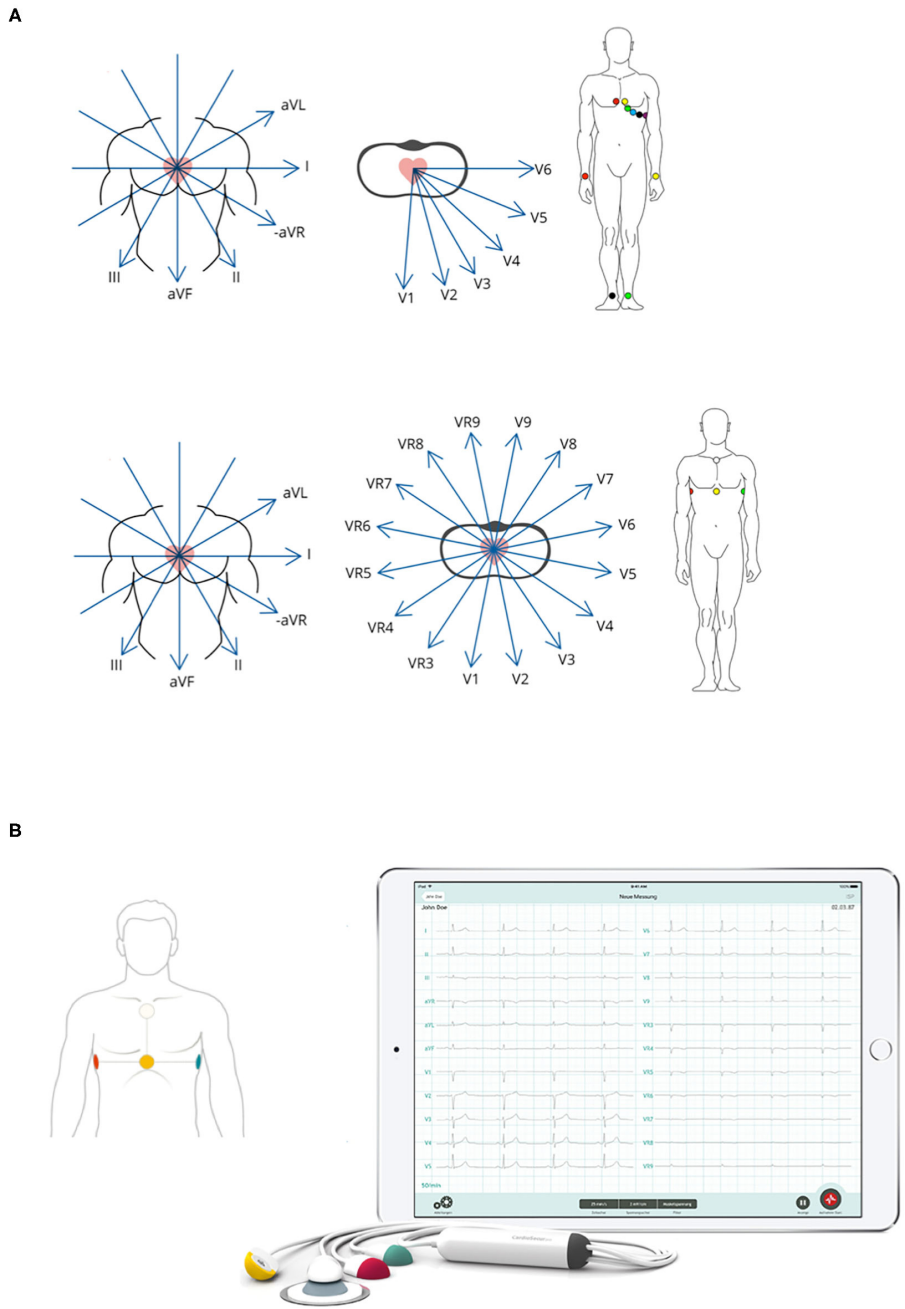
Illustration of c12L-ECG and CS-ECG electrode placement and derived leads. **(A)** Respective positioning of necessary electrodes and resulting leads are illustrated for both c12L-ECG (top panel) and CS-ECG (bottom panel) acquisition. **(B)** Picture of the CS-ECG system as used during the present trial. Illustrations courtesy of Personal MedSystems GmbH.

The CS system used for this study consists of a 4-electrode ECG cable connected to a tablet computer equipped with the respective CS software module (CardioSecur Pro Version 2.4.0–2.5.4). Placement of the 4 CS electrodes was performed at predefined positions according to the manufacturer's protocol: cranial and caudal end of patient's sternum as well as horizontally (to the caudal sternal electrode) at the left and right medio-axillary line (Figure 1). C12L-ECG systems, generally combination systems with integrated defibrillators, manufactured by Zoll (mostly X-Series), or Corpuls (C3) were used as available on scene. Placement of electrodes was performed according to standard procedure and guidelines with acquisition of additional leads (right ventricular and posterior) left to the treating team's discretion (Figure 1).

### Selection of Participants and Data Acquisition

Respective teams were instructed to obtain parallel recordings of c12L-ECG and CS-ECG tracings for patients who were subjected to triage for suspected ACS. All ECGs were sent digitally to receiving hospitals whenever technically feasible.

In compliance with the study protocol and approval by the ethics committee, teams were explicitly instructed to proceed with standard care without delay, i.e., not to let CS-ECG acquisition protract any therapeutic interventions deemed necessary and time-critical by treating teams. Patients were transferred to hospitals with catheterization capacities and patients were treated according to standard ACS algorithms and standard operating procedures. On hospital arrival, an intrahospital c12L-ECG (± additional posterior and right-precordial leads) was recorded and treatment continued per standard hospital protocols.

Final hospital reports including definitive diagnoses, all intra- and extrahospital ECG tracings, results of cardiac catheterization and laboratory tests were obtained from the patients' hospital records after discharge or death.

Importantly, comprehensive ECG interpretations by prehospital teams were not documented and therefore not available for comparison of diagnostic accuracy. Thus, evaluation of diagnostic accuracy only compares retrospective interpretation of acquired prehospital ECG tracings (both c12L-ECG and CS-ECG) by blinded cardiologists with final hospital records (the latter serving as the “diagnostic gold standard” with which both prehospital recordings/ECG modalities were retrospectively compared).

Retrospective readings of CS-ECG and c12L-ECG were compared in terms of detection of clinically relevant major ECG changes (qualifying for STEMI-treatment) in the prehospital emergency setting (2). Furthermore, these STEMI diagnoses were correlated with the need for percutaneous coronary interventions (PCI).

Treating emergency physicians and their teams were asked to evaluate the CS-ECG system with a standardized questionnaire (please refer to Supplemental Figure 1). Each physician was advised to complete a single questionnaire, preferably after treatment of their first patient but otherwise once they felt that they could answer all questions adequately. Matching of individual questionnaires with specific patients was not intended and not possible due to the anonymous nature of the questionnaire. Questions covered estimations of diagnostic yield, speed and safety of the CS-ECG. Furthermore, user-friendliness, general applicability and feasibility as well as physician-perceived patient comfort were assessed in comparison with current c12L-ECG systems.

Generally, manual aspects such as actual application in terms of electrode placement, cleaning/rigging and data transmission were taken care of by paramedics (mandated minimum training of 3-years in prehospital EMS), while aspects such as ECG interpretation and diagnostic/therapeutic decisions were completely left to the EP. Regarding EPs' level of expertise and rank, all physicians in this study had completed at least 3 years of training of their respective subspeciality (anesthesia, cardiology, gastroenterology or hematology) and had been board-certified as emergency physicians (having completed mandatory minimum in-hospital training of 2 years, at least 6 months of ICU experience, a curriculum of supervised prehospital training by experienced EPs as well as comprehensive classes on prehospital emergency medicine).

Importantly, treating physicians on scene were responsible for completion and data integrity of the questionnaire. In this regard, they were advised to weigh and integrate feedback of their team members for/into their judgement, whenever they felt this to be necessary and/or helpful for their evaluation of the CS system. Furthermore, participating EPs had been advised to complete the questionnaire with their team members present for a more comprehensive evaluation, if possible.

Ratings for system-related and technical issues ranged from “very complicated” to “very easy.” Ratings with “rather, predominantly, or very easy” were considered positive in terms of CS implementation for grouped analyses, while “partially complicated/easy” was considered neutral and “rather and very complicated” were judged as negative statements.

Statements regarding physician- and patient-centered issues such as estimations of diagnostic performance and potential therapeutic implications were rated by degree of approval (ranging from “completely disagree” to “completely agree”). Here, calculation of overall positive assessment and endorsement of the CS system by emergency physicians was deducted from all statements rating CS implementation as “rather, predominantly, or completely agree.” Agreement to some extent was considered a neutral voting, whereas “rather or completely disagree” were considered to be negative evaluations.

The study protocol was approved by the institutional review board and ethics committee of Heidelberg University (S-378/2015), and written informed consent was obtained from all patients or their legal representatives.

### Statistics

Quantitative variables are reported as medians with interquartile range, while absolute and relative frequencies are given for categorical data. Wilcoxon signed-rank test was used for comparison of c12L-ECG and CS-ECG systems in terms of physician preference and perceived diagnostic improvement. Sensitivity, Specificity, positive predictive value (PPV) and negative predictive value (NPV) were calculated for both c12L-ECG and CS-ECG systems to evaluate the diagnostic performance. *P* < 0.05 were considered to indicate statistical significance. Statistical analyses were performed with “R” (Version 3.4).

## Results

A total of 203 patients were included in the current study. Median age of study participants was 67 years (interquartile range: 55–79 years) with 135 (67%) patients being male (Table 1).

**Table 1:** Baseline characteristics and distribution of study participants.

Total patient number, *n*			203
Age, years—median (IQR)			67 (55–79)
Male Gender, *n* (%)			135 (67%)
Prehospital triage, *n* (%)	Suspected STEMI	c12L-ECG CS-ECG	57 (28%) 65 (32%)
Cardiac catheterization, *n* (%)			88 (44%)
Percutaneous coronary intervention (PCI), *n* (%)			60 (30%)
Final hospital diagnosis, *n* (%)	Non-cardiac origin		53 (26%)
	cardiac origin (non-ischemic) ACS		56 (28%)
		Total	94 (46%)
		STEMI	34 (17%)
		NSTEMI	30 (15%)
		UA/CHD	30 (15%)

*IQR, interquartile range; STEMI, ST-elevation myocardial infarction; c12L-ECG, conventional 12-lead electrocardiogram; CS-ECG, CardioSecur-electrocardiogram; ACS, Acute Coronary Syndrome; NSTEMI, Non-ST-elevation myocardial infarction; PCI, percutaneous coronary intervention; UA, Unstable Angina; CHD, Coronary Heart Disease*.

According to hospital records 64 (31.5%) patients had myocardial infarction. Out of these 64 patients a total of 34 patients were actually diagnosed with STEMI and total vessel occlusion in coronary angiography according to final hospital reports.

Cardiac catheterization was performed in 88 (43.3%) patients and percutaneous coronary interventions (PCI) were performed in 60 (29.6%) patients.

### Comparative Qualitative Evaluation of c12L-ECG and CS-ECG Systems

**Figure 2:**
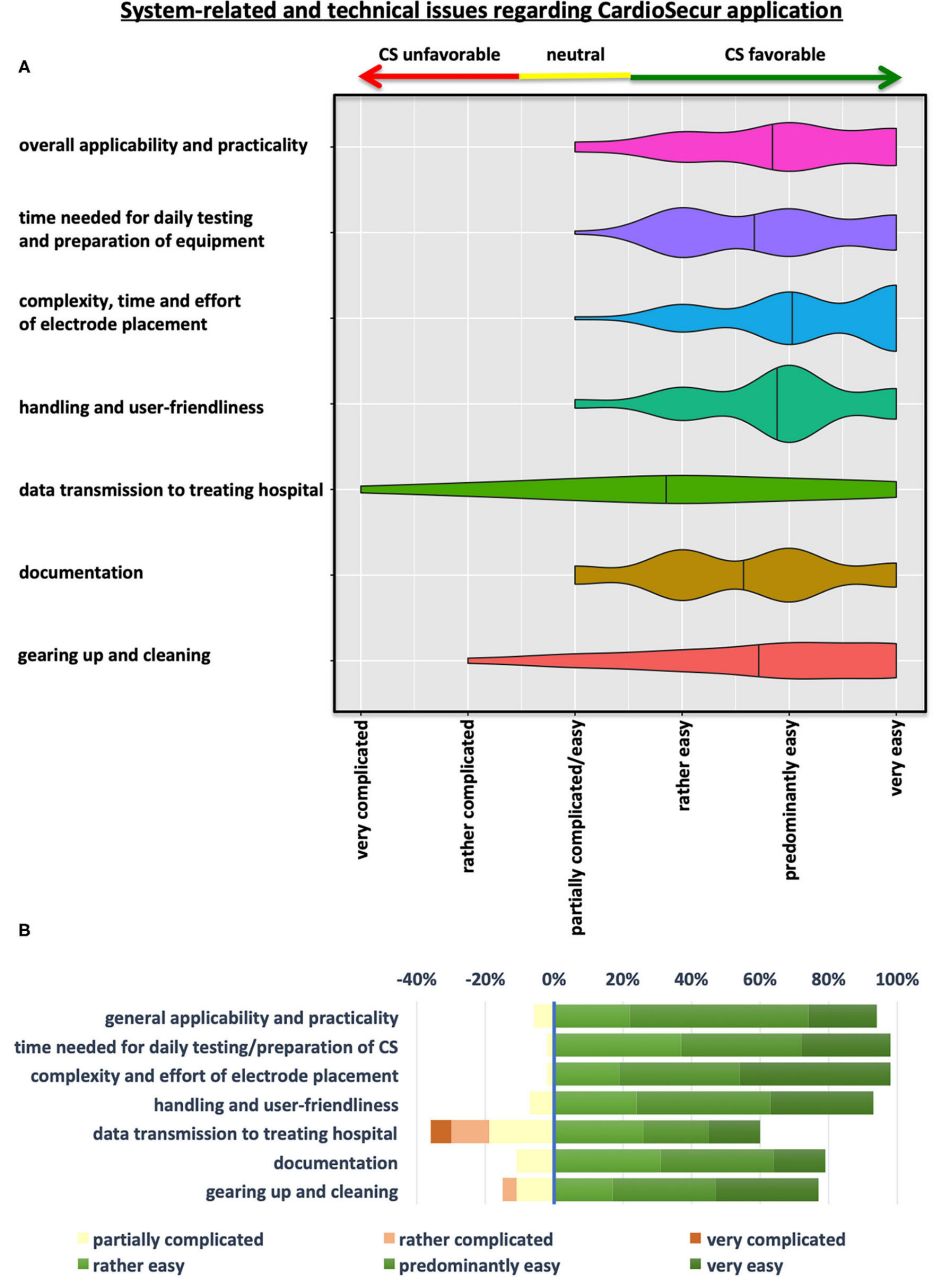
System-related and technical issues regarding CS-ECG application. **(A)** Violin Plots depicting the results of the questionnaire on system-related and technical aspects of CS implementation in prehospital emergency settings. **(B)** Illustration showing a grouped-analysis of positive, neutral and negative emergency physicians' ratings concerning various aspects of CS application. Ratings with “rather, predominantly, or very easy” were considered positive in terms of CS implementation for grouped analyses (green colors), while “partially easy/complicated” and “rather and very complicated” were judged as neutral (yellow) or negative (shades of red) statements, respectively.

Overall, 54 treating emergency physicians and their teams completed the standardized questionnaire. The first part of the standardized questionnaire provided data on system-related and technical issues of CS application as perceived by emergency response teams (Figure 2 and Table 2).

**Table 2:** System-related and technical issues regarding Cardio-Secur application.

**Statements regarding use of the CS-ECG system**	**Very** **complicated**	**Rather** ** complicated**	**Partially complicated**	**Rather** ** easy**	**Predominantly** ** easy**	**Very** ** easy**
Overall applicability and practicality	0%	0%	3 (6%)	12 (22%)	28 (52%)	11 (20%)
Time needed for daily testing and preparation of equipment	0%	0%	1 (2%)	20 (37%)	19 (35%)	14 (26%)
Complexity, time and effort of electrode placing	0%	0%	1 (2%)	10 (19%)	19 (35%)	24 (44%)
Handling and user-friendliness	0%	0%	4 (7%)	13 (24%)	21 (39%)	16 (30%)
Data transmission to treating hospital	3 (6%)	6 (11%)	10 (19%)	14 (26%)	10 (19%)	8 (15%)
Documentation	0%	0%	6 (11%)	17 (31%)	18 (33%)	8 (15%)
Gearing up and cleaning	0%	2 (4%)	6 (11%)	9 (17%)	16 (30%)	16 (30%)

*Statements regarding application/implementation of the CS-ECG system were assessed by the questionnaire with ratings for system-related and technical issues ranging from “very complicated” to “very easy.” Tabular view of the results of the questionnaire with absolute numbers representing the count of answers by EPs in this category followed by the respective fraction (in %) in brackets*.

The majority of users attested easy general applicability and practicality of the device (93%) as well as user-friendliness in terms of handling and application of CS systems in emergency situations (94%) (Figure 2 and Table 2). Time, effort and complexity of electrode placement were perceived as easy by almost all teams (98%). Furthermore, time needed for daily testing and preparation of equipment were judged favorably for CS by almost all emergency physician teams (98%). Simplicity of documentation (80%) as well as cleaning and rigging of equipment after usage (76%) were also perceived as easy by most emergency teams. Advance data transmission to destination hospitals received mixed judgements with problems encountered by 35% of treating teams. Yet, a majority of physicians (59%) still rated data transfer to respective facilities to be simple (Figure 2 and Table 2). As EPs and their teams could write comments on individual positive or negative aspects of the device and its handling, we can deduct from these that data transmission was often compromised—mainly due to lack of network coverage (only about 2/3 of Germany are currently covered by the 4G standard).

### Evaluation of Diagnostic Performance and Therapeutic Implications of CardioSecur®

**Figure 3:**
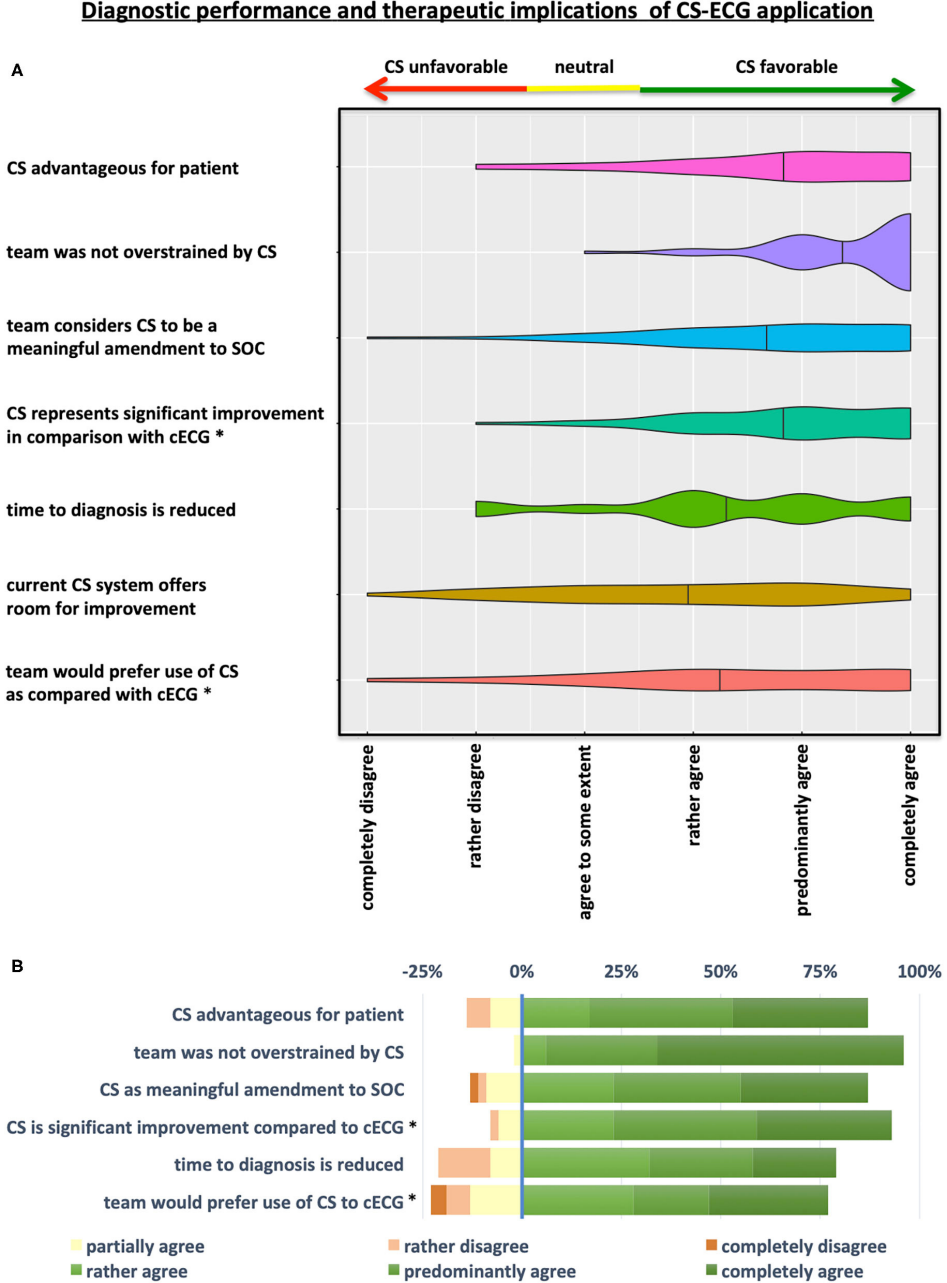
Diagnostic performance and therapeutic implications of CS-ECG application. **(A)** Violin Plots depicting the results of the questionnaire on diagnostic and therapeutic aspects of CS implementation in prehospital emergency care. **(B)** Illustration showing a grouped-analysis of positive, neutral and negative ratings concerning various diagnostic and therapeutic implications of CS application by degree of approval. Calculation of overall positive assessment and endorsement of the CS system by emergency physician-led teams was deducted from all statements rating CS implementation with “rather, predominantly, or completely agree” (shades of green). Agreement to some extent was considered a neutral voting (yellow), whereas “rather or completely disagree” were considered to be negative evaluations (shades of red). *p < 0.001 for comparison CS-ECG vs. conventional 12-lead ECG (cECG).

The second part of the standardized questionnaire featured statements related to physician-perceived diagnostic performance and therapeutic implications of CS application as compared to standard of care c12L-ECG recordings (Figure 3 and Table 3). The majority of emergency medical professionals (77%) declared that they generally preferred application of CS to c12L-ECG (*p* < 0.001) with even more teams (87%) estimating CS to be a meaningful amendment to standard of care (Figure 3 and Table 3). These findings were substantiated by the perception that CS represents a significant improvement in comparison with c12L-ECG (*p* < 0.001 for superiority of CS vs. c12L-ECG) for nearly all participants (92%).

**Table 3:** Diagnostic performance and therapeutic implications of CS-ECG application.

**Statements regarding use of the CS-ECG system**	**Completely** **disagree**	**Rather** **disagree**	**Partially** **agree**	**Rather** **agree**	**Predominantly agree**	**Completely agree**
CS advantageous for patient	0%	3 (6%)	4 (8%)	9 (17%)	19 (36%)	18 (34%)
Team was not overstrained by CS	0%	0%	1 (2%)	3 (6%)	15 (28%)	33 (62%)
Team considers CS to be a meaningful amendment to SOC	1 (2%)	1 (2%)	5 (9%)	12 (23%)	17 (32%)	17 (32%)
CS represents significant improvement in comparison with cECG	0%	1 (2%)	3 (6%)	12 (23%)	19 (36%)	18 (34%)
Time to diagnosis is reduced	0%	7 (13%)	4 (8%)	17 (32%)	14 (26%)	11 (21%)
Current CS system offers room for improvement	1 (2%)	7 (13%)	11 (21%)	11 (21%)	15 (28%)	6 (11%)
Team would prefer use of CS as compared with cECG	2 (4%)	3 (6%)	7 (13%)	15 (28%)	10 (19%)	16 (30%)

*Statements regarding physician- and patient-centered issues such as estimations of diagnostic performance and potential therapeutic implications were rated by degree of approval (ranging from “completely disagree” to “completely agree”). Tabular view of the results of the questionnaire with absolute numbers representing the count of answers by EPs in this category followed by the respective fraction (in %) in brackets*.

While most operators (60%) agreed that the current CS system left room for further improvement, there was broad consensus that teams were not overstrained by CS operation and implementation (96%). Statements by EPs in the free text field of the questionnaire indicate that—apart from the desire to hold a print-out version of the CS-ECG—longer, and thus more convenient CS electrode cables as well as improvement of the software (more comfortable patient data entry) are the main ideas for optimization.

When asked about patient-centered issues, a broad majority voiced agreement that time to diagnosis is reduced by implementation of CS (79%). Moreover, 87% of teams considered implementation of CS to be advantageous for patients when compared to standard of care c12L-ECG (Figure 3 and Table 3).

Interestingly, 15% of participants reported that CS-ECG resulted in diagnostic or therapeutic ramifications in comparison with c12L-ECG. In congruence, 3 of 34 STEMI patients in our study collective were only recognized by CS-ECG tracings and were missed during prehospital triage by standard 12-lead c12L-ECG recordings.

### Quantitative Evaluation of Diagnostic Results of Prehospital c12L-ECG and CS-ECG Recordings

Prehospital c12L-ECG and CS-ECG tracings had identified 31 (91.2%) patients with STEMI and total coronary vessel occlusion each (Supplemental Table 1). Interestingly, apart from one case our data showed that no additional leads had been recorded by any physician team as part of prehospital ACS-management. Overlap of patients congruently diagnosed with STEMI in both modalities was observed in 28 patients (82.4%). Each system missed three of 31 STEMI cases detected by the other modality (yielding a detection rate of 90.3% for cross comparison). Moreover, further analyses of these six discrepant cases, in which one ECG modality seems to have failed, demonstrated a median time interval of 20 min (range: 4–45 min) between both recordings. As expected and in congruence with the study protocol based on the recommendations by the ethics committee, c12L-ECG tracings had been recorded prior to CS-ECG leads in all 6 cases.

When STEMI diagnoses in either prehospital c12L-ECG or CS-ECG recordings were analyzed in terms of need for percutaneous coronary intervention, sensitivity and specificity for c12L-ECG were 0.63 and 0.87, while CS-ECG provided a sensitivity of 0.7 and a specificity of 0.84. Positive predicitive value (PPV) was 0.67 for c12L-ECG and 0.65 for CS-ECG; calculation of negative predictive value (NPV) yielded 0.85 and 0.87, respectively (Supplementary Figure 2).

## Discussion

Prehospital management of ACS patients remains challenging due to limited resources, logistical difficulties and urgency of risk stratification forcing time-critical decisions (2, 8). This predicament is aggravated by lack of prehospital diagnostic tools to corroborate respective decisions (8). In this regard, the present study contributes first experience with CS-ECG, a simplified ECG system which calculates even more leads than the standard c12L-ECG (22 vs. 12 leads) despite requiring less electrodes to be placed (four instead of at least 10 electrodes).

This first feasibility study showed that implementation of a CS-ECG system was feasible and user-friendly in the prehospital emergency setting both in terms of technical issues and in terms of practicality and applicability.

In this time- and logistically-limited setting, the fast and convenient placement of electrodes is of critical importance, which the vast majority of teams rated as straightforward and easy with the CS system. In support of this evaluation, our data showed that—apart from one case—no additional leads for c12L-ECG had been recorded by any emergency physician, although strict posterior STEMI is often masked when only using c12L-ECG, and additional leads derived from additional electrodes (V7 - V9) may be required for accurate diagnosis. In this regard, 3 STEMI patients in our study collective had not been identified by standard 12-lead ECG acquisition and may have benefitted from additional leads.

Congruently, most teams felt that time to diagnosis could be accelerated significantly by CS implementation, which they even perceived as conveying a significant benefit for the patient's further diagnostic and therapeutic pathway, as 9% of STEMI patients in our trial were only identified as such by supplemental CS-ECG recordings during prehospital triage. On the basis of these data and free text commentaries it is tempting to speculate, that the therapeutic ramifications perceived by a number of EPs in our study may indeed derive from this combination of (subjective) improvements in terms of precision and speed of diagnostic algorithm (e.g., leading to improved recognition of strictly posterior MI).

Evidently, these subjective assessments of a potential (time) benefit deserve further evaluation by future studies.

Furthermore, it seems logical that emergency staff generally favored application of CS-ECG over c12L-ECG, especially since comfort of CS-ECG in terms of preparation of the system, on-scene application as well as post-treatment maintenance (cleaning and re-rigging) were consistently rated to be easy by team members. This is an important factor whenever implementation of innovative methods is attempted, since analyses show that acceptance of new methods significantly depends on perceived benefit for patients as well as simplicity of handling and application (20, 21). This holds especially true for prehospital interventions and algorithms when time matters critically and teams tend to set high value on efficiency, simplicity and suitability for daily use. In line with this train of thought, emergency teams suggested potential for further optimization of the CS system in terms of advance data transmission (most likely due to bad network coverage in rural areas) as well as optimization pertaining to the prehospital emergency setting (longer cables, fast entry of patient data, attachable protective case).

Since the main objective of this trial pertained to user perceptions regarding diagnostic performance and therapeutic implications of CardioSecur^®^ as well as system-related and technical issues of CS application in the prehospital emergency setting, this study was not designed and powered to evaluate non-inferiority or superiority of the CS-device as compared to c12L-ECGs. However, the accuracy and predictive performance of EASI-/vector-based ECG calculations have been analyzed in multiple trials over the past decades with accumulated data demonstrating non-inferiority in comparison to conventional gold-standard 12-lead ECG tracings in various intrahospital settings (16–19, 22–27). Reassuringly, data of this study show that detection of STEMI-patients by CS-ECG was similar when compared to gold-standard c12L-ECG algorithms, though sensitivity did not reach 100% with either system alone. This finding leaves room for speculation with two possible reasons coming to mind. Firstly, positioning and acquisition of both c12L-ECG and CS-ECG may not have been perfect, resulting in decreased precision and sensitivity of traces. Especially, clinically unstable and agitated patients may have impaired diagnostic performance of the acquired ECGs. Secondly and likely more importantly, electrocardiographic findings in ACS patients may show dynamic alterations over time and in some cases the exact timing of ECG acquisition may be of critical importance, especially in light of other prehospital therapeutic interventions such as aspirin, heparin and morphine (8, 28–30).

Logistic reasons as well as individualized decisions by treating emergency physicians appear to have caused delays (in CS-ECG acquisition) in some instances. Congruently, in those 6 STEMI patients with discrepant findings between both recordings a median time interval of 20 min between c12L-ECG and CS-ECG acquisition was observed. This probably resulted from emergency physicians having been advised by the study protocol (as mandated by the institutional review board) that no time-critical decisions and treatments were to be impaired by study conduction and protracted by CS-ECG acquisition. This stipulation may also provide an explanation for the relatively small number of critical ACS patients included in the present study.

Overall, both ECG systems had similar sensitivity, specificity as well as PPV and NPV in our study collective. When prehospital STEMI diagnoses by either system were retrospectively matched with the need for PCI during the following hospital stay, 63% of c12L-ECG patients classified as STEMI during prehospital triage received PCI treatment, while positive STEMI criteria in CS-ECG even resulted in a sensitivity of 70% in terms of necessity of PCI. An ongoing trial for comparative analysis of c12L-ECG and CS-ECG recordings during ergometric studies will assess diagnostic non-inferiority on a broader basis (31). Moreover, trials with simultaneous CS-ECG and c12L-ECG acquisitions in the intrahospital setting in emergency departments as well as coronary care units may augment this data. Obviously, doubtless demonstration of diagnostic non-inferiority of the CS-ECG system by large prospective multicenter trials will be a sine qua non before CS-ECG may actually be considered for simplification of prehospital ECG acquisition.

Therefore, currently available data from this pilot study show that safety of diagnostic algorithms does not seem to be impaired by simplification of ECG acquisition with CS systems in prehospital emergency patients.

## Study Limitations

Limitations of this study include the single-center setting and the limited sample size.

While subjective evaluation by emergency physicians suggests accelerated care and logistics by CS-ECG, objective data on these perceived advantages is lacking. Furthermore, patient perception of CS-ECG was not studied in this trial, although it appears reasonable to believe that patient-perceived comfort will be significantly higher with CS-ECG.

Furthermore, favorable ratings by early adopters are not necessarily representative of the wider intended users.

Despite the effort to acquire ECG tracings of both systems at the same time, simultaneity in ECG acquisition was not achieved in all study participants, in that c12L-ECGs were generally recorded first, as c12L-ECGs had often been acquired by first-responders and paramedics even before the physician-staffed team with the CS-system arrived on scene. As the institutional review board had required that trial conduction was not to impair or protract diagnostic or therapeutic algorithms, treating EPs and their teams probably waived repeated/parallel ECG acquisition of c12L-ECGs in case these had already been acquired. However, due to dynamic alterations in ACS patients over time, the exact timing of ECG acquisition may be of critical importance (8, 28–30). Future comparative studies will have to ensure correct electrode placement, synchrony and longitudinal diagnostic non-inferiority in this regard.

## Conclusion

According to this first feasibility study in the prehospital emergency setting, implementation of a CardioSecur^®^ ECG system confers benefits in terms of user-friendliness and practicality of ECG acquisition. While exploratory analyses suggest diagnostic yield of CS-ECG to be similar to c12L-ECG recordings, treating teams voiced a significant preference of CS-ECG over c12L-ECG systems and felt CS-ECG to accelerate and significantly improve emergency care of ACS patients.

## Data Availability Statement

The raw data supporting the conclusions of this article will be made available by the authors, without undue reservation.

## Ethics Statement

The studies involving human participants were reviewed and approved by Institutional review board and ethics committee of Heidelberg University, Heidelberg, Germany. The patients/participants provided their written informed consent to participate in this study.

## Author Contributions

SS was responsible for study design, performed data collection and analyses, interpretation, and drafted the manuscript. HK and JS were involved in data collection and assisted with analyses. MG contributed to data analyses and interpretation of all statistical analyses. TZ, EP, and HK were involved in study design, implementation and analyses. MP was responsible for study design, implementation and analyses, and is corresponding and guarantor author. All authors contributed to the development of the manuscript and approved its content prior to submission.

## Conflict of Interest

The authors declare that the research was conducted in the absence of any commercial or financial relationships that could be construed as a potential conflict of interest.

## Supplementary Material

Supplemental Figure 1 Evaluation of the CardioSecur-ECG system with a standardized questionnaire. Physicians were asked to complete this questionnaire on system-related and technical issues as well as diagnostic and therapeutic implications of CS-ECG application.

Supplemental Figure 2 Diagnostic performance of c12L-ECG in comparison with CS-ECG. Prehospital STEMI diagnosis: Sensitivity, specificity, positive predictive value (PPV) and negative predictive value (NPV) regarding need for PCI. Sensitivity: c12L-ECG 0.63, CS-ECG: 0.7.

Supplemental Table 1 Exploratory analysis of diagnostic performance of c12L-ECG vs. CS-ECG tracings. Top panel compares prehospital STEMI diagnoses (c12L-ECG and CS-ECG retrospectively analyzed by blinded cardiologists) with intrahospital STEMI diagnoses based on hospital records (occluded coronary vessel + ECGs) as gold standard, while middle panel compares these (retrospective) prehospital STEMI diagnoses with need for PCI. Bottom panel: comparison of prehospital repolarization abnormality with need for PCI. STEMI, ST-elevation myocardial infarction; PCI, percutaneous coronary intervention. ^*^1 Diagnosis based on blinded retrospective analysis of respective prehospital ECG. ^*^2 Diagnosis based on final hospital records.

## Abbreviations

ACS: Acute Coronary Syndrome
cECG: conventional electrocardiogram
CS: CardioSecur^®^
CS-ECG: Cardio-Secur^®^ electrocardiogram
c12L-ECG: conventional (10-electrode/12-lead) electrocardiogram
ECG: electrocardiogram
NPV: negative predictive value
NSTEMI: Non ST-elevation myocardial infarction
PCI: percutaneous coronary intervention
PPV: positive predictive value
STEMI: ST-elevation myocardial infarction
UA: Unstable Angina (Pectoris).
