# Supplementary figures and images for: Feasibility of CardioSecur®, a Mobile 4-Electrode/22-Lead ECG Device, in the Prehospital Emergency Setting
Source: Front Cardiovasc Med. 2020 Oct 9;7:551796. doi: 10.3389/fcvm.2020.551796 (PMC7581708; doi:10.3389/fcvm.2020.551796)

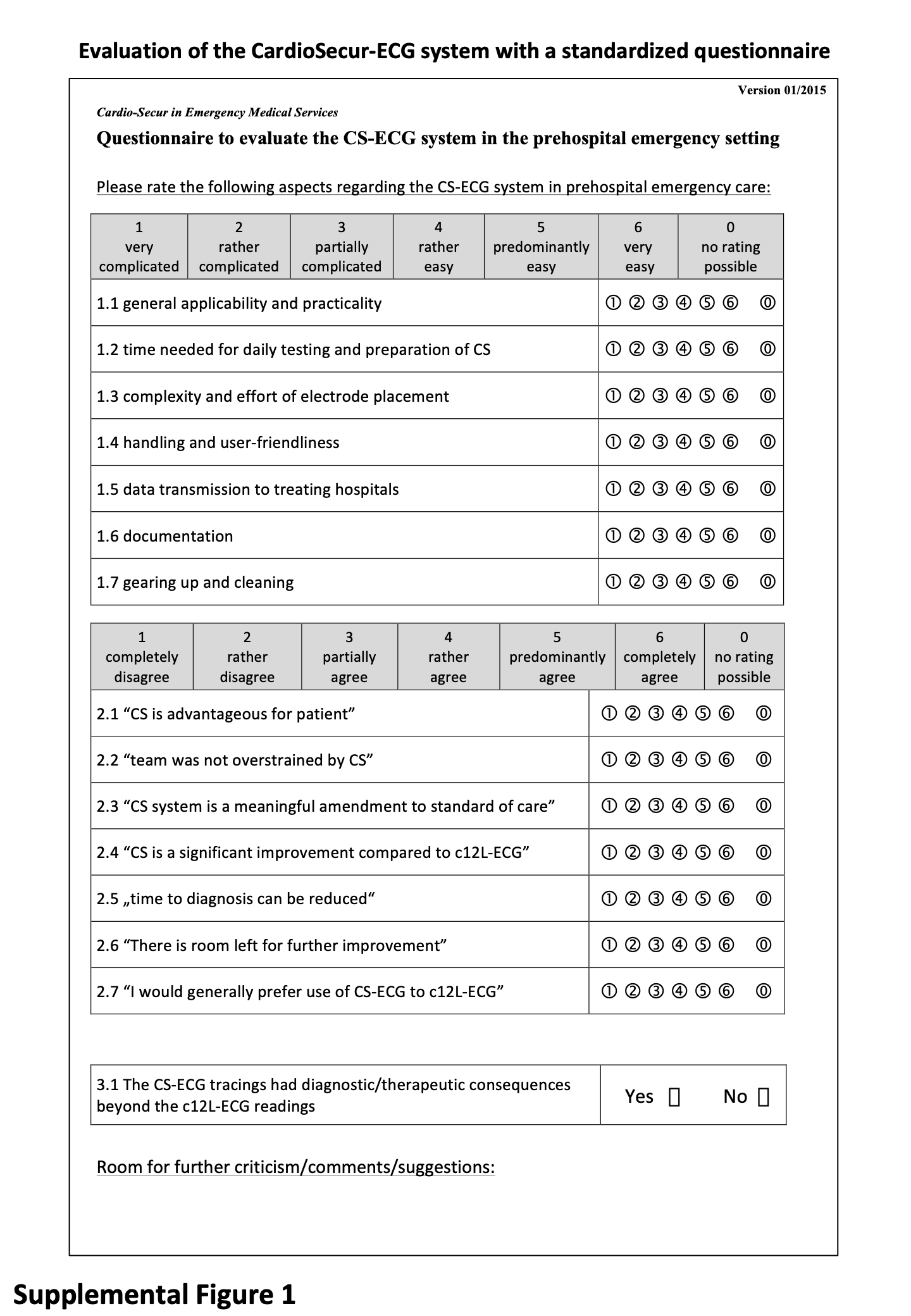

Supplement: Supplemental Figure 1 — Evaluation of the CardioSecur-ECG system with a standardized questionnaire. Physicians were asked to complete this questionnaire on system-related and technical issues as well as diagnostic and therapeutic implications of CS-ECG application. [file Image_1.TIFF]

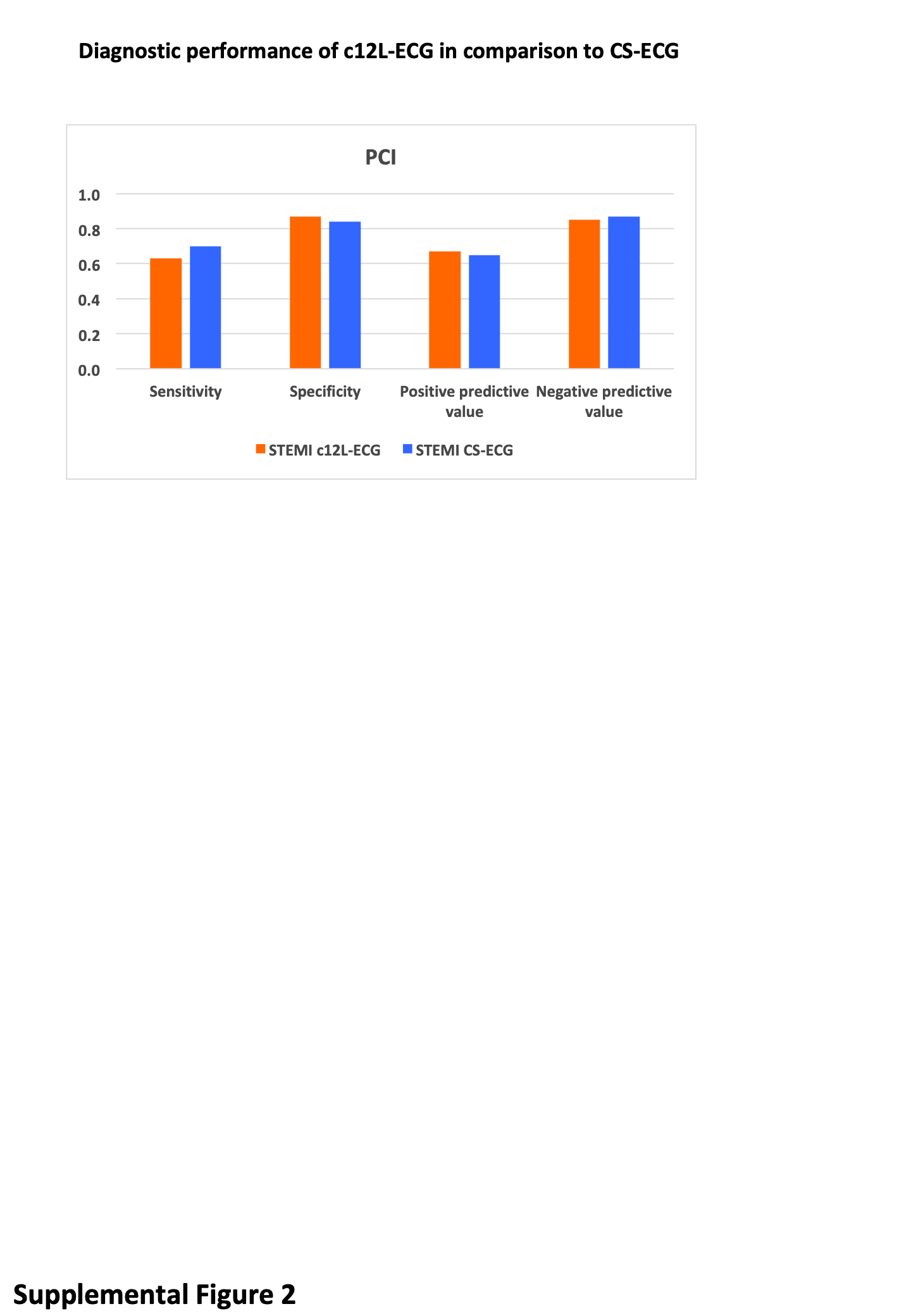

Supplement: Supplemental Figure 2 — Diagnostic performance of c12L-ECG in comparison with CS-ECG. Prehospital STEMI diagnosis: Sensitivity, specificity, positive predictive value (PPV) and negative predictive value (NPV) regarding need for PCI. Sensitivity: c12L-ECG 0.63, CS-ECG: 0.7. [file Image_2.TIFF]
