# Supplementary material for: Feasibility of CardioSecur®, a Mobile 4-Electrode/22-Lead ECG Device, in the Prehospital Emergency Setting
Source: Front Cardiovasc Med. 2020 Oct 9;7:551796. doi: 10.3389/fcvm.2020.551796 (PMC7581708; doi:10.3389/fcvm.2020.551796)
Supplement: Supplemental Table 1 — Exploratory analysis of diagnostic performance of c12L-ECG vs. CS-ECG tracings. Top panel compares prehospital STEMI diagnoses (c12L-ECG and CS-ECG retrospectively analyzed by blinded cardiologists) with intrahospital STEMI diagnoses based on hospital records (occluded coronary vessel + ECGs) as gold standard, while middle panel compares these (retrospective) prehospital STEMI diagnoses with need for PCI. Bottom panel: comparison of prehospital repolarization abnormality with need for PCI. STEMI, ST-elevation myocardial infarction; PCI, percutaneous coronary intervention. *1 Diagnosis based on blinded retrospective analysis of respective prehospital ECG. *2 Diagnosis based on final hospital records. [file Table_1.DOCX]

**Supplemental Tables:**

**Supplemental Table 1. Exploratory analysis of diagnostic performance of c12L-ECG vs CS-ECG tracings**

| **STEMI *^1^** | | **c12L-ECG** | **CS-ECG** |
| --- | --- | --- | --- |
| **STEMI *^2^** | **Sensitivity** | **0.91** | **0.91** |
|  | **Specificity** | **0.85** | **0.80** |
|  | **Positive predictive value** | **0.54** | **0.48** |
|  | **Negative predictive value** | **0.98** | **0.98** |
| **PCI *^2^** | **Sensitivity** | **0.63** | **0.70** |
|  | **Specificity** | **0.87** | **0.84** |
|  | **Positive predictive value** | **0.67** | **0.64** |
|  | **Negative predictive value** | **0.85** | **0.87** |
|  |  |  |  |
| **Repolarization abnormalities *^1^** |  | **c12L-ECG** | **CS-ECG** |
| **PCI *^2^** | **Sensitivity** | **0.88** | **0.88** |
|  | **Specificity** | **0.50** | **0.51** |
|  | **Positive predictive value** | **0.42** | **0.43** |
|  | **Negative predictive value** | **0.91** | **0.91** |

**Supplemental Table 1. Exploratory analysis of diagnostic performance of c12L-ECG vs CS-ECG tracings.** Top panel compares prehospital STEMI diagnoses (c12L-ECG and CS-ECG retrospectively analyzed by blinded cardiologists) with intrahospital STEMI diagnoses based on hospital records (occluded coronary vessel + ECGs) as gold standard, while middle panel compares these (retrospective) prehospital STEMI diagnoses with need for PCI. Bottom panel: comparison of prehospital repolarization abnormality with need for PCI.

Abbreviations: STEMI (ST-elevation myocardial infarction); PCI (percutaneous coronary intervention)

**1 Diagnosis based on blinded retrospective analysis of respective prehospital ECG*

**2 Diagnosis based on final hospital records*
